# Editorial: Exploiting non-oncogene addiction for overcoming drug resistance in metastatic tumors

**DOI:** 10.3389/fphar.2026.1798268

**Published:** 2026-02-26

**Authors:** Albrecht Reichle, Dennis Christoph Harrer, Florian Lüke, Tobias Pukrop, Lina Ghibelli, Daniel Heudobler

**Affiliations:** 1 Department of Internal Medicine III, Hematology and Oncology, University Hospital Regensburg, Regensburg, Germany; 2 Division of Personalized Tumor Therapy, Fraunhofer Institute for Toxicology and Experimental Medicine, Regensburg, Germany; 3 Bavarian Cancer Research Center (BZKF), University Hospital Regensburg, Regensburg, Germany; 4 Department of Biology, University of Rome “Tor Vergata”, Rome, Italy

**Keywords:** coatomer subunit zeta-1, integration of oncogene-driven information in tissue systems, anakoinosis, non-oncogene addictions, pioglitazone (Pio), receptor-triggered transcription factors, systems pharmacology, tumor tissue editing

Acquired genetic aberrations implement new information about how tissue systems should perform (Mueller et al. 2018; Hendrix et al. 2007). Emerging tumor systems integrate oncogene-driven information by coordinating vast networks of non-oncogenes within tumor and heterogeneous adjacent stroma cells, ultimately organizing non-oncogene addiction (NOA)-driven circuitries to ensure the viability and resilience of malignant tissues (Xu et al. 2024; Blazquez et al. 2020).

Integrated oncogenic-triggered information, promoting NOA genome editing, becomes conceivable in novel tissue structures and functions. However, phenomenology does not reveal how oncogenic input is integrated transcriptionally, particularly with regard to the operation of novel oncogene-driven NOA circuitries that span tumor tissue systems (Yuan et al. 2024).

The reproducibility of tumor phenomenology, which facilitates traditional tumor classification, hypothesizes that similar integration of oncogenic data is associated with a distinct input of oncogenic information. However, how is oncogenic information integration formally structured at the non-oncogene level?

All five papers focus on the tension between oncogenic information input and comprehensive integration of non-oncogenes.

Di Marco describes Coatomer subunit zeta-1 (COPZ1), a protein exhibiting characteristics of NOA in multiple tumor types. Its expression may be normal or increased compared to non-malignant cells. COPZ1 is genome-agnostically integrated across many tumor histologies (Di Marco et al.). Knockdown of COPZ1 leads to tumor cell death in various neoplasias. Silencing COPZ1 can involve Golgi damage, blocking autophagy or the unfolded protein response, silencing the type I interferon pathway, or activating ferroptosis. Although COPZ1 is differentially integrated into tumor systems’ NOA circuitries among different tumor histologies, uniform biological effects occur when targeted (Di Marco et al.).

The paper by Jadhav highlights a functionally heterogeneous integration of N-myc downregulated gene 1 (NDRG1) as NOA protein upon differential oncogenic information input. NDRG1 acts either as a facilitator (triple-negative breast cancer) or an inhibitor of tumor progression (Jadhav et al.; López-Tejada et al. 2023; Deng and Des Richardson 2023). NDRG1 mutations are rare and mostly missense (Saponaro et al. 2025). NDRG1 is involved in cancer hallmarks, interacts with the oncogenic protein p53, and plays a role in promoting tumor expansion (Joshi et al. 2022).

Li et al. approached the Research Topic of how to disturb oncogenic information integration with bile acids and their respective derivates serving as ligands of the nuclear receptors FXR and PXR (Li et al. 2024; Fiorucci et al. 2024; Fu et al. 2019). Bile acids are important in regulating lipid, carbohydrate metabolism, but also immune system and inflammation (Fiorucci et al. 2024).

Bile acids play a dual role in malignant cells, acting either as tumor promoters of esophageal, colon cancer and cholangiocarcinoma or as tumor growth inhibitors in many tumor histologies *in vitro* (Zimber and Gespach 2008; Yang et al. 2025). Bile acids regulate the interfaces between cell proliferation and differentiation, cell death versus survival, invasion and metastasis, processes that are obviously guided context-dependently. Like many nuclear receptors, bile acid receptors are rarely mutated and are involved in regulating cancer proliferation (Gangwar et al. 2022; Renga et al. 2012).

Tumor tissue editing (TTE) has emerged as an effective method/template of incorporating receptor-triggered transcription factors involved in the integration of oncogenic data into tumor therapy. This novel approach enables successful control of relapsed or refractory (r/r) metastatic neoplasias (Harrer et al. a).

**FIGURE 1:**
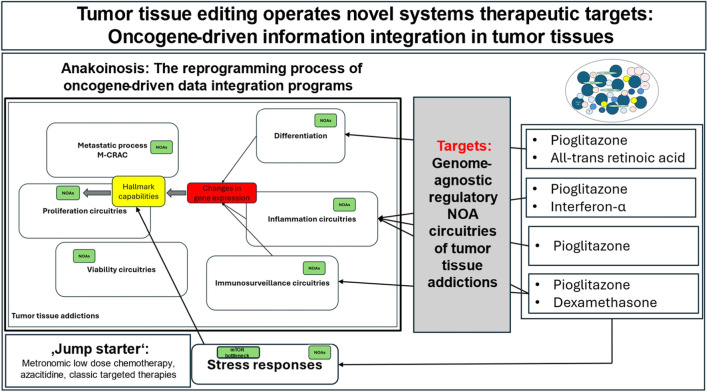
Oncogenic data integration in NOA circuitries is a genome-agnostic process and histologically different tumors may share accessibility for disturbing data integration process by tumor tissue editing. M-CRAC = post-therapy metastasis, cancer cell recolonization, acquired resistance and genetic heterogeneity; NOA = non-oncogene addiction. Adapted form Harrer et al. a, licensed under CC BY.

TTE, combined receptor-triggered editing of both cancer and stroma cells by pioglitazone, a PPARα/γ agonist, with or without dexamethasone, all-trans retinoic acid or interferon-α, alongside the simultaneous promotion of tumor stress responses with ‘jump starters’ that interfere with transcriptional data integration, i.e., metronomic low-dose chemotherapy, azacitidine and classic targeted therapies (such as everolimus and lenalidomide), clinically facilitates the reprogramming and deconstruction of the oncogene-triggered transcriptional integration machinery constituted by NOAs and NOA circuitries (Figure 1) (Solimini et al. 2007; Luo et al. 2009; Harrer et al. a).

TTE-initiated reprogramming of NOA circuitries may be followed by pivotal responses in r/r hematological malignancies, cancers and sarcomas, as indicated by long-term disease stabilization, objective response, complete remission (CR) or continuous CR. Transcriptionally targeting oncogene-driven data integration plus ‘jump starter’ may resolve heterogeneous intra-tumor resistance. The data suggests that novelly arising NOA circuitries manage oncogenic transcriptional data integration but cannot yet be comprehensively pinned down (Harrer et al. a).

According to the therapeutic success of selected TTE protocols in r/r neoplasias, NOA circuitries that provide identical patterns of clinically important receptor-triggered transcriptional targets may be present in (molecular)-genetically heterogeneous neoplasias, and differential patterns in identical tumor histologies. Notably, identical NOA integration programs have been identified across different tumor histologies, suggesting a genome-agnostic NOA accessibility altogether (Harrer et al. a).

NOA organizations are novel in that they are genome-agnostically supervised by the oncogene-driven transcriptional integration machinery, as shown in 15 clinical trials by the successful receptor-triggered transcriptional accessibility of r/r neoplasias via differential TTE techniques (Harrer et al. a; Harrer et al., 2026). Single NOAs can be pointedly edited, e.g., in r/r Hodgkin’s lymphoma for disturbing the NOA-promoted oncogenic data integration with classic targeted therapy, everolimus (Harrer et al. b). Among 13 histologically distinct metastatic r/r neoplasias, only in r/r metastatic gastric cancer data integration could not be disrupted through the addition of pioglitazone in a randomized TTE approach.

In terms of information, a tumor system creates a distinctive identity by establishing NOAs/NOA circuitries. Thus, the relation between parts and the whole is not simply a claim about a correlation between structures/functions and oncogenic information integration (Reichle und Hildebrandt 2009; Marshall et al. 2023; Di Marco et al.; Jadhav et al.). Therefore, TTE designs adapted to oncogenic data integration patterns are directed to specific systems constellations and therefore, characterized by manageable toxicities (Harrer et al. a).

Due to highly heterogeneous oncogenic events, we participate in an unbelievable number of diverse histologies. However, as indicated clinically, the integration of oncogenic data is manageable in therapeutic terms within the context of systems pharmacology. This is due to the suggested tightly structured transcriptional data integration programs and the multifold developable TTE approaches with repurposed drugs.

In contrast to the various, heterogeneous non-synonymous oncogenic events in treated r/r neoplasias, differential TTE techniques reveal that data integration programs are pivotal editable targets, are shared between neoplasias, and genome-agnostic (Alexandrov et al. 2013; Vogelstein et al. 2013; Harrer et al. a).

Current research does not focus on reconstructing the mechanistic basis of oncogenic data integration programs (Hendrix et al. 2007; Johnson et al. 2026). One clue to their mechanisms of action is the restoration of tumor plasticity among cancer hallmarks and their reprogramming profiles following TTE. This includes the induction of differentiation, control of inflammation, enhancement of immunosurveillance, metabolic reprogramming and compromising tumor viability (Lowe et al. 2004; Harrer et al. a). The process of transcriptional reprogramming of data integration programs, which restores therapeutically valuable plasticity rather than resistance development, is called anakoinosis (Harrer et al. a).

Oncogenic data integration-driven NOA reorganization turned out to be an instrumental target for TTE techniques, representing a novel promising systems pharmacological approach. In the future, diagnosing NOAs, NOA circuitries, oncogenic-integrated receptor-triggered transcription factors and editability of NOAs may be as valuable for selecting therapy as histological/molecular genetic tumor typing is for establishing personalized hematology/oncology.
